# COVID-19 Place Confinement, Pro-Social, Pro-environmental Behaviors, and Residents’ Wellbeing: A New Conceptual Framework

**DOI:** 10.3389/fpsyg.2020.02248

**Published:** 2020-09-01

**Authors:** Haywantee Ramkissoon

**Affiliations:** ^1^School of Business & Economics, Faculty of Biosciences, Fisheries & Economics, UiT The Arctic University of Norway, Tromsø, Norway; ^2^College of Business, Law & Social Sciences, Derby Business School, University of Derby, Derby, United Kingdom; ^3^College of Business & Economics, Johannesburg Business School, University of Johannesburg, Johannesburg, South Africa

**Keywords:** COVID-19, place confinement, pro-environmental and pro-social behavior, place attachment, habits, residents’ wellbeing, behavior change, health

## Abstract

Residents’ wellbeing in the present COVID-19 global health crisis requires a deeper understanding to determine appropriate management strategies to promote sustainable behaviors and contribute to human and planetary health. Residents’ behavior can have a profound influence in contributing to personal and global community’s health by responding effectively to emergency strategies in disease outbreaks such as the Coronavirus. It is evident that an understanding of residents’ behavior(s) pre COVID-19 across fields have relied on over-simplistic models, many of which will need to be revisited. Our interaction with people and nature while respecting social distancing has profound positive impacts on our physical, mental, emotional, and spiritual wellbeing. The current health pandemic has called that people be confined in their homes across many nations as a means to control the spread of the virus and save lives. This calls for research exploring the mechanisms; this paper develops and proposes a conceptual framework suggesting that place confinement promotes pro-social and household pro-environmental behaviors which could become habitual and contribute further to our people’s and our planet’s health. The proposed model concerns socio-economically favored social sectors whose survival is not as threatened by the pandemic as the poor sectors where wellbeing (and mental health) are at high risk. Some evidence shows that human connectedness to place may contribute to engagement in desirable behaviors. Interaction with other members of the household can help create meanings leading to collective actions promoting psychological wellbeing. Promoting hygienic behaviors in the household (frequent hand washing) while at the same time being conscious not to keep the water flowing when not required would contribute to a range of benefits (health, financial, biospheric, altruistic) and promote wellbeing. Engaging in pro-social behaviors may result in positive effects on psychological wellbeing, reducing mental distress giving rise to a sense of attachment and belongingness, trust and overall life satisfaction. Engaging people in low-effort pro-environmental behavior to maintain some levels of physical activity and biological harmony with natural environmental settings (e.g., gardening) may help reduce anxiety and distress. This is the first study exploring the interplay of relationships between place confinement, pro-social behavior, household pro-environmental behaviors, place attachment as a multi-dimensional construct and presenting their relationships to residents’ wellbeing. Behavioral change interventions are proposed to promote lifestyle change for people’s wellbeing and broader societal benefits.

## Introduction

Residents’ wellbeing in the present COVID-19 global health crisis requires a deeper understanding to determine appropriate management strategies to promote sustainable behaviors and contribute to human and planetary health. Residents’ behavior can have a profound influence in contributing to their personal and the global community’s health, by responding effectively to emergency strategies in disease outbreaks such as the Coronavirus. It is evident that an understanding of residents’ behavior(s) pre COVID-19 across fields have relied on over-simplistic models, many of which will need to be revisited. The pandemic is calling for more research into health and wellbeing of the global community requiring efficient and long-lasting behavioral change (Betsch et al., 2020).

Across many nations, the COVID-19 pandemic has demanded that people spend time in their home settings and limit their essential travel within their communities to control the spreading of the SARS-COV-2 virus. This has been proclaimed to be an essential measure to put in place to limit the spread of infection protecting the global population’s health. While measures of social distancing have been effective in managing the significant pressures on health services, researchers argue confining people in their homes are having negative consequences on human health (Douglas et al., 2020; Holmes et al., 2020). Scholars have been advocating the health benefits of human to human interactions since a very long time (see House et al., 1988a, b). In addition to the significant role our social connections play in promoting wellbeing (Ramkissoon et al., 2018), engaging in activities keeping us connected to nature while being confined in our place also has important benefits for mankind (Hartig et al., 2014; Ulmer et al., 2016).

In an attempt to address impacts of social distancing on residents’ health and physical and mental wellbeing, this paper develops and proposes a conceptual framework suggesting that place confinement can provide a context that may promote pro-social and household pro-environmental behaviors which could become habitual and contribute further to both our people’s and our planet’s health. The proposed model concerns socio-economically favored social sectors whose survival is not as threatened by the pandemic as the poor sectors where wellbeing (and mental health) are at high risk. Some evidence shows that human connectedness to place may contribute to engagement in pro-social and pro-environmental behaviors (e.g., De Dreu et al., 2015; Rosa et al., 2018). Interaction with other members of the household can help create meanings leading to collective actions promoting their psychological wellbeing. For instance, promoting hygienic behaviors in the household (e.g., frequent hand washing) while at the same time being conscious not to keep the water running for longer than required can be promoted in households. With the global pandemic threatening the globe, individuals may be motivated by fear. The need to utilize place confinement as a context to engage in desirable target behaviors is even more pronounced. If collectively adopted by members of the household, it can generate a number of benefits (health, financial, biospheric, altruistic) and contribute to individual, collective (in the household) and societal wellbeing.

A recent study by Lin et al. (2020) suggests that increased search for washing hands on Google was correlated with a lower spread of COVID-19 in a sample across 21 countries. This adds support to the current study; collective meanings being created in COVID-19 through public awareness has potential to promote the “frequent hand washing” behavior across nations. The author argues our individual and collective choices will influence our future. Pro-social and pro-environmental behaviors need to be targeted and promoted within the household, and at the local, national and global levels through shared values (to save lives and protect our planet). Promoting healthy behaviors brings a range of societal and environmental benefits and encourage collegial support from family and friends to help foster collective actions. With much uncertainty revolving around the global pandemic, researchers are of the view that adopting individual and collective behaviors will help reduce the spread of the virus and protect lives (Anderson et al., 2020). The author argues that interventions need to be designed and implemented. Place confinement provides a window of opportunity setting the context for new habits creation and habits reinforcement for behavioral change. It is further important to identify the barriers and enablers that will guide the choice of these interventions for residents’ wellbeing. The author further argues that development of interventions has to be drawn from theory and practical issues encountered by people confined to their environmental settings. It is important to first design the content of interventions based on desired target behaviors and identify how the designed interventions will be delivered. Interventions will need to be feasible and relevant to context.

There’s increasing evidence that engaging in pro-social behaviors in the home environment (e.g., discussing health and safety with family) may result in positive effects on psychological wellbeing, reducing anxiety (e.g., McCarroll et al., 2009; Padilla-Walker et al., 2015) and improving the residents’ wellbeing and overall quality-of-life. Further, engagement in individual and collective pro-environmental behaviors gives rise to a sense of attachment and belongingness, trust and satisfaction (e.g., Nunkoo et al., 2012; Ramkissoon et al., 2012). In the present COVID-19 context, engaging people in low-effort household pro-environmental behavior (Ramkissoon et al., 2013a) to maintain some levels of physical activity and biological harmony with the natural environmental settings may help reduce anxiety and distress, hence promoting one’s wellbeing. There is growing evidence that engaging in pro-environmental behaviors contributes to health and wellbeing (e.g., Townsend et al., 2018; White et al., 2019) lending further support to using place confinement as a context for individuals to engage in more environmentally sustainable household behaviors.

Bringing together multi-disciplinary evidence from environmental, social and cognitive psychology, political science, tourism, behavioral and public health literature, this study develops and proposes a new conceptual framework to critically examine how residents’ place confinement can serve as a positive pathway to make them become more place attached through their engagement in pro-social and household pro-environmental behaviors. The model is developed adopting a coherent and integrated approach to advance knowledge and contribute to real life impacts. This paper critically examines the context of place confinement and its likely influence on residents’ pro-social behavior and pro-environmental behavior. The model further proposes that promoting pro-social and pro-environmental behaviors among residents positively influence their place attachment with their home and community’s environmental settings which is fundamental to their wellbeing and nature’s protection. This is the first study to explore the interplay of relationships between place confinement, pro-social behavior, household pro-environmental behaviors, place attachment as a multi-dimensional construct (with sub-dimensions of place dependence, place identity, place affect and place social bonding) and presenting their relationships to residents’ wellbeing. The proposed single integrative model makes an important contribution to place confinement and place attachment literature, pro-social, pro-environmental behavior and wellbeing literature.

Aligning with COVID-19 research calls to promote global health, promoting pro-social and pro-environmental behaviors in the household can provide preventive and sustainable measures to improve public health and wellbeing. This paper provides important insights to policy makers, discussing how behavioral change interventions need to be increasingly adopted to promote lifestyle change. This will require new directions to reinforce the broader benefits of habit change for both people’s wellbeing and planetary health. The study’s implications further align with the 2030 Agenda for sustainable development in safeguarding our planet’s resources and promoting healthier communities post the global health pandemic.

## Literature Review

### Place Confinement

People across the globe have been talking about being “place confined” for the past few months. This has been since nations enforced mobility restrictions to curb the rapid spread of the global health pandemic SARS-COV-2 which started in Wuhan, China in December 2019. The simplistic definition of place confinement will be physical immobility, where people are restricted to their place of residence, community, state, or country. While most governments have imposed place confinement, there are a few exceptions which did not enforce strict confinement measures. An example of the latter is Sweden in contrast to its Nordic neighbors, Denmark, Finland and Norway which had closed their borders earlier on during the pandemic. As of this date 14 May 2020, no COVID-19 vaccine has been produced. While confinement measures are gradually relaxing in a number of countries, there are still many nations which remain in total lockdown.

The term “confinement” has been explored in anthropology literature and mostly studied in specific contexts including hospitals, homes for the elderly, prisons and detention centers (e.g., Chapman et al., 2006; Vandentorren et al., 2006). In Appadurai’s (1988) conception of place confinement, natives of a place could also feel confined in a place until they move to a new place. While it might seem that they have escaped the confinement, Appadurai (1988) argue they have simply escaped to a new place where they could equally start feeling confined. This suggests that place confinement can also be conceptualized as perceived threat to mobility.

Evidence from health literature indicates that when confinement restricts individuals’ physical activities, this may be perceived as a lack of independence among people impacting on their mental health (e.g., Haney, 2003; Metzner and Fellner, 2013). This has been commonly noted in healthcare of the elderly where staff could actually be doing more harm than good to the elderly. This happens when staff overly engage in assisting the elderly in activities such as dressing and eating, the latter may become dependent and less determined to make efforts (Doumit and Nasser, 2010) which can impact further on their mental and physical wellbeing. The dependence of elderly people in homes may be a result of institutionalization which may be very different from those confined in their own place of residence. Lack of physical activity among prisoners due to isolation punishment has also been linked with panic disorders and anxiety (Arrigo and Bullock, 2008; Mears and Reisig, 2006).

Place confinement in epidemics has not been widely investigated with the exception of a few studies prior to COVID-19 (e.g., Zhu et al., 2017; Orset, 2018). The notion of being place confined during epidemics can also bring a sense of isolation and emotional distress (Aronson, 2002). Since the virus outbreak, there has been increasing debates on place confinement and mental health issues. Some evidence in extant literature show that people being confined during epidemic outbreaks may not readily adopt confinement behaviors in the first instance, but they subsequently do so after being quarantined or due to having increased self-awareness (Zhu et al., 2017) which may help in supressing the spread of the epidemic.

Physical mobility has been significantly decreased for many as a result of the confinement rules across nations, leading to other health issues. Having some degree of physical activity remains important and has been reported to have a positive influence on individuals’ physical and mental wellbeing (Rejeski and Mihalko, 2001). It is being strongly encouraged that people stay physically active within their home environment through home exercises and other household activities. Walks in the neighborhood and parks are now allowed in some countries with COVID-19 measures of social distancing respected (e.g., Honey-Roses et al., 2020).

### Pro-social, Pro-environmental Behaviors and Place Attachment

The COVID-19 outbreak has triggered individual and collective changes, people have had/are having to change their behaviors with the change in context. This is known as “habit discontinuity” providing a window of opportunity for behavior change (Verplanken et al., 2018). Individuals’ habits may be temporarily blocked requiring alternative habits (Jones and Ogilvie, 2012); one such example is having to be place confined to prevent the spread of the SARS-COV-2 virus. Individuals have had to re-orientate and find new ways of doing things. An important reason why people may change their behavior in a habit discontinuity context is as a response to fear fueling their need to reconsider some important goals and values (e.g., Verplanken et al., 2018), which for many in the current pandemic context is their health and safety.

Protection Motivation Theory (PMT) (Rogers, 1975) conceptualized as an individual’s response to a fear appeal has been applied mostly in health sciences where fear is used to motivate change in health behaviors. Researchers have since applied PMT in environmental and social psychology literature (Gardner and Stern, 2002; Van Zomeren et al., 2010). With its focus on threat and perceived efficacy, Protection Motivation Theory recognizes that individuals will take action if they perceive a serious threat (e.g., the fear of contracting and spreading the COVID-19 virus) and they feel capable of engaging in behaviors that will make a difference (e.g., staying at home and maintaining physical distancing to avoid the spread of the virus). Drawing from PMT, providing messages targeted at triggering fear alone will be unsuccessful, strong fears will trigger highest behavioral change only when people feel a strong sense of efficacy (Ramkissoon and Smith, 2014; Van Bavel et al., 2020). Thus, it follows that interventions designed in the place confinement context need to clearly inform individuals about the benefits and consequences of non-adherence to new habits. This has potential to guide adoption of the desired behaviors and eventually become a habitual routine.

COVID-19 rising concerns on the spread of the virus, increasing mortality rates, lack of medical facilities in some countries and growing anticipation for the COVID-19 vaccine continue to raise important questions for medical practitioners, behavioral scientists, government, policy makers, businesses and the general public. This paper explores an important question: how a change in habitual household behaviors could be more beneficial for people’s wellbeing during and post the COVID-19 pandemic? This is an important question to address as it has important implications for achieving key wellbeing goals in public health in the global community.

Literature evidences that when there is a significant change in context (e.g., Bamberg, 2006; Padilla-Walker et al., 2015), people may be required to re-think and devise new ways of doing habitual behaviors. This requires the need to adopt a mindset change and adapt to contextual changes. Having been place confined in their homes (and neighborhoods) has required adoption of new behaviors. People are having to re-consider their usual household behaviors and engage in “new behaviors” linked to important values. These newly adopted behaviors may in turn become part of their self-identity (Verplanken and Sui, 2019) and be maintained over a long period of time.

COVID-19 has provided a window of opportunity for people to break and engage in habits which have a range of benefits not only for people’s health but also for planetary health. Human health and the health of our planet are intrinsically connected. Researchers across disciplines have been calling for sustainable consumption of the planet’s resources (Whitmee et al., 2015; Myers, 2017), failing which we will be further endangering our societies and planet Earth. People often unconsciously engage in behaviors posing a threat to themselves and others. This was argued in a scientific magazine a century ago on lessons from the Spanish flu (Soper, 1919). The current global Coronavirus situation demands that we take urgent action to protect both human and planetary health by mitigating the devastating environmental impacts of human consumption.

COVID-19 place confinement habit discontinuity is an opportunity to reconsider our old habits and consumption behaviors and redefine our lifestyle. The break in our routines and unhealthy behaviors provides a basis for longer term maintenance of new behaviors. As argued, adoption of pro-social and pro-environmental behaviors in a COVID-19 confinement context can lead to creation of new habits and long-term behavior change. Pro-social behavior can be examined in terms of how one person can help another, at the interpersonal level and also collectively (Vollhardt, 2009). Literature evidences that people behave pro-socially based on their peers’ behaviors; this is known as conditional pro-social behavior. People engage in the latter because they want to conform to a social norm (Messick, 2000), or they want to be fair (reciprocity) (Rabin, 1993). Pro-social behaviors have been largely applied in social psychology and has had a surge of interest from researchers across other disciplines.

Pro-environmental behavior (PEB) finds its roots in environmental psychology literature and has been adapted to a range of disciplines including geography, urban planning and architecture, tourism, health, and sociology. PEB is defined as the adoption of behaviors by individuals or groups to promote environmental sustainability (Ramkissoon et al., 2013c). People also create collective meanings leading to engagement in pro-environmental behaviors in shared settings. For behavioral change to be effective, the newly adopted pro-social and pro-environmental behaviors have to be repeated in a stable context over time (Klöckner and Matthies, 2012). Habits are slow to change; new habits have to be created. Habit formation requires that individuals get the opportunity for practice, along with other mechanisms to keep them motivated during the new habit formation process (Verplanken and Wood, 2006). When a behavior is new, untried and unlearnt, the behavioral intent is responsible for adoption of the behavior (Triandis, 1977). However, as the behavior occurs and becomes habitual, habits become a better predictor of behavior (Triandis, 2005). Place confinement in COVID-19 provides the context to practice a new habit(s).

Drawing on extant literature on pro-social behaviors and the recent COVID-19 multidisciplinary research (e.g., Beck and Hensher, 2020; Brooke and Jackson, 2020; Li et al., 2020; Rajkumar, 2020), the current study proposes some examples of pro-social behaviors in a COVID-19 context namely: discussion with family related to health and safety; collective home exercises to maintain health; visit to the supermarket respecting social distancing; collective decision on eating habits to support health; maintain a good online social network; regular meaningful conversations with the elderly. Household pro-environmental behaviors can include water-saving behaviors (using half-flush, having shorter showers, closing the tap when brushing teeth/shaving, closing the tap soon after washing hands); recycling; home composting, food waste avoidance (e.g., Curtis et al., 2013; Ramkissoon et al., 2015). Interventions through communication and engagement will be needed during the place confinement context for adoption and reinforcement of pro-environmental behaviors over time.

Householders’ self-efficacy and personal cost-benefits are personal-level predictors of pro-environmental behaviors (Klöckner, 2013). Once the behavior is adopted, the new habit will develop as the individual repeats the same behavior in the same situation over time and gets rewarded for it (e.g., health, financial, biospheric, altruistic benefits). As people are likely to maintain the new habits when they see the efficacy of their behaviors, this could foster long term behavior change and promote place attachment.

Place attachment is defined as the emotional bond people share with environmental settings (Ramkissoon, 2015). The concept originates from place attachment theory (Bowlby, 1962) depicting the mother-infant bond, and how this relationship evolves to other social and environmental settings. Place attachment has been extensively studied across disciplines including psychology, sociology, geography, tourism, environmental management and health (e.g., Attale and Consoli, 2005; Rosenbaum et al., 2009; Jiang et al., 2017). Place dependence and place identity remain the two prominent sub-constructs of place attachment. Researchers however have argued that place attachment consists of other sub-dimensions; for instance, Ramkissoon et al. (2012) argue place affect and place social bonding as two other sub-dimensions of place attachment in addition to place identity and place dependence.

### Place Attachment and Wellbeing

A way to support people’s wellbeing in the current health crisis and post the pandemic is through place attachment. Drawing on Ramkissoon’s and colleagues’ work on place attachment (Ramkissoon et al., 2013b, c), this study proposes the four sub-dimensions of place attachment: place dependence, place identity, place affect and place social bonding have a positive influence on residents’ wellbeing. Place dependence refers to how best a place fulfills an individual’s desired outcomes. Place identity refers to the meanings associated and sense of belongingness one feels with a place. Place affect has been described as the emotional bonding an individual forms with a place. Place social bonding is characterized as social bonds that are formed in a place (see Ramkissoon, 2015).

Literature evidences that social involvement and individuals’ low and high-effort pro-environmental behaviors positively influence place attachment (e.g., Stedman, 2002; Ramkissoon and Mavondo, 2015, 2017; Ramkissoon et al., 2018). People engaging in these behaviors may get more emotionally attached to their home/community environments which may in turn influence their wellbeing. This is further supported in behavioral medicine literature (e.g., Attale and Consoli, 2005). Dependence on a place’s distinctive attributes has been shown to positively influence residents’ wellbeing. People may get more dependent on their home settings, e.g., their gardens, which may fulfill their functional purpose and they would not want to change their place for another (Stokols and Shumaker, 1982; Halpenny, 2010). There is a need to identify the barriers and enablers that will guide the choice of interventions to foster place dependence. In the COVID-19 context, people may develop a stronger sense of dependence on the physical settings of their home environments and may no longer feel mentally confined and isolated as their levels of dependence with their place increase since the latter is fulfilling their goals. Examples include they could get place dependent on their home gym and might no longer need a gym membership. People could start enjoying home-made food and might no longer want to go back to regular take-away meals. An increasing pool of research have debated how regular take aways contribute to poorer health (e.g., Lui and Ndzogoue, 2018; Grunseit et al., 2019) and calling for food habits behavior change. Adopting these new behaviors have important health and wellbeing as well as financial benefits (Lee and Loke, 2005; Mackay et al., 2017).

An additional benefit is also the use of place confinement as a pathway for fostering place social bonding which can subsequently contribute to wellbeing. People in confinement settings could start developing a stronger sense of self-identity with their home and recognize that it is distinctive from other places. Place identity reinforces sense of wellbeing, bringing a sense of security and “feel good” factor (Williams and McIntyre, 2012). While we are being told to maintain social distancing which has led us to fear how we can be harmed by or harm significant others, with appropriate interventions, individuals can learn to strengthen their social bonds with loved ones while being place confined. The crisis communication messaging on social bonding is particularly important (e.g., Brooke and Jackson, 2020; Public Health England, 2020) as in the midst of the pandemic, people can experience tensions between themselves. This has important implications e.g., couples, parents, children and those living with the elderly can further develop their social bonds while respecting social distancing rules when place confined and coping with the crisis. We can keep our social ties by talking to our neighbors and going for short walks where permissible while maintaining social distancing. Place social bonding was found as a significant predictor of residents’ quality of life in Australia (Ramkissoon et al., 2018) with people connecting with others and creating shared meanings contributing to their overall life satisfaction.

Since the origin of the SARS-COV-2 virus, there’s been continuous discussion on mental wellbeing of populations. The health emergency calls for place social bonding measures to be implemented for residents’ wellbeing. Public Health England (2020) published guidelines on maintaining the elderly’s wellbeing during the pandemic. Other initiatives in the UK have encouraged neighbors and communities to communicate while maintaining social distancing (Brooke and Jackson, 2020). Researchers in China report psychological interventions (e.g., Duan and Zhu, 2020) to assist with mental health issues.

Our attachment has evolved to encompass social relations (Bowlby, 1988), the fear of losing touch with family and friends can cause mental distress and isolation. The author recommends that people continue to maintain their social connections in the home, community, and online social networks while respecting enforcement laws and social distancing measures. This can help reinforce people’s attachment security (Schimmenti et al., 2020) and assist in developing COVID-19 fear coping strategies.

The pandemic has also reinforced people’s social networks over the internet (Thelwall and Thelwall, 2020), people exchange information with shared meanings for a common purpose and important benefits. Examples include people respecting confinement rules, staying home and maintaining social connections using internet. This helps in preventing the spread of the virus, assessing COVID-19 anxiety through telemedicine, and providing loneliness therapies for the elderly aging in place through internet-based cognitive behavior therapy (e.g., Armitage and Nellums, 2020; Hernández-García and Giménez-Júlvez, 2020; Ohannessian et al., 2020; Smith et al., 2020; Ting et al., 2020). These studies lend further support to the author’s argument: social bonding is a pro-social behavior and has important health and wellbeing outcomes for people in confined spaces. Exchanging information has a range of benefits including information dissemination, increased awareness levels (Majeed et al., 2020) of pandemic threats and observing the efficacy of collective behaviors.

COVID-19 has been shown to be causing mental health problems requiring governments to implement effective measures. The World Health Organization (2020) has called for strategies to be developed to provide psychosocial considerations. Promoting place affect which is about developing the emotional bond that a person shares with his/her place and its settings (Ramkissoon, 2016) can be used as an effective tool providing psychological support to people during this pandemic and after. Place affect has been researched in the public health domain and shown to have a positive influence on psychological restorativeness and wellbeing (Townsend et al., 2018). Developing a sense of place affect, where one feels a deep bonding to one’s home environment or specific settings of the home and community during COVID-19 place confinement and post pandemic, could contribute to a sense of peace and calmness and meet wellbeing goals. Some evidence in literature shows that people may embrace spirituality when a disaster strikes (Captari et al., 2019); place affect can hence contribute to spiritual wellbeing. Researchers argue spiritual wellbeing continues to attract significant interest (Counted and Zock, 2019; Counted et al., 2020) in contributing to residents’ overall quality of life.

**FIGURE 1:**
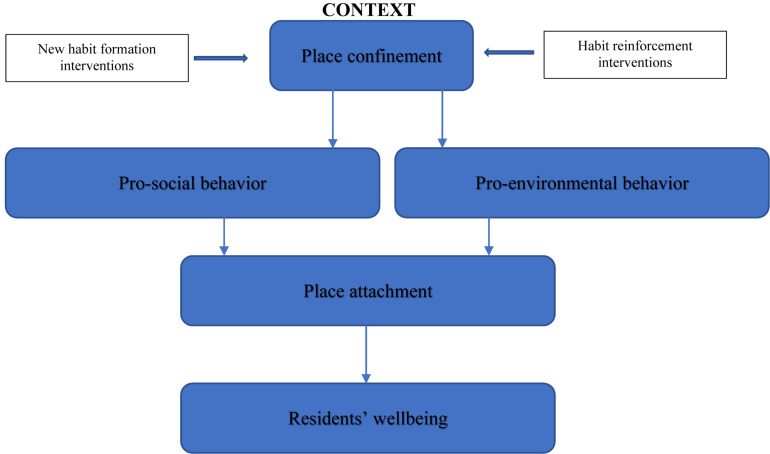
The proposed theoretical framework.

The above discussion shows that our interaction with people and nature (while respecting social distancing measures) during place confinement can have profound positive impacts on our physical, mental, emotional, and spiritual wellbeing. This paper draws on multidisciplinary literature (e.g., Van Zomeren et al., 2010; Ramkissoon et al., 2018; Van Bavel et al., 2020) and develops and proposes a single integrative model of residents’ wellbeing. The associations between the constructs under investigation are depicted by arrows in the conceptual framework (see Figure 1).

## Discussion and Conclusion

### Formation of New Habits

It is crucial to consider how these new pro-social and pro-environmental behaviors are developed and sustained. The challenge is to achieve long term behavioral change. First, effective interventions will be required to change habitual behaviors in the households and community to create and promote new long-lasting healthy behaviors. In many ways, our behaviors are habitual, we do not undergo an elaborate reasoning (Verplanken et al., 2018). It is very much about what we have learnt, these bits of information are stored in our memory and the more we perform a behavior, the more we associate to the information retrieved from our internal memory search resulting in our habitual behaviors (Orbell and Verplanken, 2010; Ramkissoon and Nunkoo, 2012). People repeatedly performing a behavior in a specific context, implicitly link their stored information in memory with the context and responses. In the current COVID-19 context, this will require the learning of a new habit(s) (Lally et al., 2010; Egner, 2014). The significance of having context cues is discussed.

Our habitual behavior may tend to be selective on information cues that confirm our behavioral choice and disregard what does not conform to our choices. People usually act when they see the efficacy of their behaviors. Context cues need to include efficacy messages focusing on how the target pro-social and pro-environmental behaviors are of benefit to users. Crisis communication social media messages can emphasize a range of health benefits, financial benefits from savings in the household, contributions to help save the planet (biospheric benefits) and contributions to improve one’s wellbeing and quality of life and that of loved ones and the broader society (altruistic benefits through individual and collective contributions by engagement in pro-social and pro-environmental behaviors). Maintaining the new behavior(s) will be important for effective long-term behavior change. The author further argues that it is important to promote a sense of local, national, and global collective identity through public communication messages showing how a collective approach through engagement in the recommended pro-social and pro-environmental behaviors is benefiting the planet and its people. Messages also need to be targeted to encourage the global community to help those suffering in isolation (e.g., the elderly, and children and adolescents missing the physical contact with their friends) through meaningful collective actions to make them feel loved and cared for.

Drawing on Diffusion of Innovation (DOI) theory (see Rogers, 2003), each person in the household can modify the adopted pro-social behavior and pro-environmental behavior(s) according to what best suits him/her. The desired goal of the target pro-social and pro-environmental behavior(s) for health, financial, biospheric or/and altruistic benefits could activate the mental representation of an individual’s habitual behavioral choice and could potentially reinforce his/her habits. Long term behavior change can be enhanced when it is customized to the individual (Rogers, 2003).

For behavioral change to be effective, the new behavior has to be repeated in a stable context over time (Klöckner and Matthies, 2012). The comprehensive action determination model (CADM) (Klöckner and Blöbaum, 2010) has been used and applied across behavioral domains to measure habit strength (e.g., Ofstad et al., 2017; Joanes et al., 2020). Aligning with the Theory of Planned Behavior (TPB) (Ajzen, 1991), the model postulates that individual environmentally relevant behavior is determined directly by intentions and perceived behavioral control and integrates habit strength as the third predictor of behavior. Prior studies have drawn on the above theories to achieve desired pro-environmental behaviors (e.g., Walton and Hume, 2011; Richler, 2017). The author argues that CADM can also be applied to achieve pro-social behaviors in both the current and post pandemic contexts.

Some behaviors may require effortful thought and could be low in automaticity. On the other hand, other behavior(s) may be much easier to undertake.

This study suggests that given that highly habituated behaviors can become automatic, it may be worth concentrating on those behaviors, and continue to monitor habit strength (Gillebaart and Adriaanse, 2017; Verplanken and Sui, 2019) post the COVID-19 pandemic so as to know when the interventions (e.g., media communication cues) could stop with a high likelihood that the behavior would continue. It is suggested that it may be likely that the crisis communication and stronger collective identity social media messages will be needed for some time to come before individuals become habituated to the practice of pro-social and pro-environmental household behaviors to promote healthier lifestyles. This is an important step to encourage appropriate behavior change during and post the COVID-19 pandemic.

## Conclusion

COVID-19 pandemic will allow researchers to test behavior change through adoption of pro-social and pro-environmental behaviors and better equip the community with sustainable behaviors in both a confinement and post crisis context. A change in core social and environmental habits is necessary to promote healthy lifestyles and protect the planet’s health. COVID-19 is leading us to re-consider our existing behaviors with opportunities to embark on new designs for a sustainable future. Through stakeholder engagement, individuals, communities, businesses, charitable organizations, healthcare providers, and governments could work together to support their people through this global health pandemic and collectively help minimize and mitigate the negative environmental impacts on our planet. Behavior change is key to a sustainable future for mankind and planet Earth. Developing programs with health-promoting behaviors encompassing pro-social and pro-environmental behaviors can have a positive impact on psychosocial wellbeing of people.

There is much anticipation regarding how long lasting COVID-19 new behaviors will be. It is expected that people would quickly revert to normal pre-COVID 19 behaviors without targeted interventions from governments and co-actors across all areas. Local and national governments need to undertake a multi-stakeholder engagement approach (Nunkoo and Ramkissoon, 2016) and promote the benefits of how engaging in pro-social and pro-environmental behaviors in households and communities can help achieve collective goals. With COVID-19, people are further concerned about financial savings. Adoption of pro-environmental behaviors can be used as a leverage for positive economic impacts. It will be important to continue the interventions in the post pandemic context until people see the effectiveness of adoption of pro-social and pro-environmental behaviors. People are important stakeholders of our local, national and global community (Nunkoo and Ramkissoon, 2011, 2012). Thoughtful and deliberate planning of how to deal with the public in this uncertain time of the pandemic will continue to require engagement strategies. Individuals in the local and global community need to be recognized as relevant stakeholders playing an important role in curbing the spread of the virus and further contribute to global health and wellbeing through the adoption of the new behaviors as discussed.

Social media is an important tool to promote the evidence of individual and collective pro-social and pro-environmental COVID-19 behaviors, people act when they see the efficacy of their behaviors. Promoting these behaviors would provide the moral support individuals need when confronting the fear of the pandemic. Individuals are moral agents (Schimmenti et al., 2020), one’s actions will have an important consequence (positive or negative) on the society and the planet. The pandemic has certainly provided the global community an opportunity to come together as responsible citizens and engage in responsible behaviors that will contribute to individual and broader societal goals.

The proposed model should be considered as a conceptual aid on positive pathways for desired behavior change outcomes in the current pandemic rather than a rigid prescription. The focus of this study is on communication and public engagement for long lasting behavior change. Future studies could consider other types of interventions. The author recognizes that the current model is favored for those social classes whose survival are not at risk as they are able to work remotely and/or are being supported by government and co-actors. The model will need to be adjusted, refined and adapted to different socio-economic contexts. For instance, people who are unable to work from home and their subsistence is being impacted are likely to experience discomfort, which can impact negatively on their wellbeing. Swift institutional interventions will be required first to help people coordinate and survive before they can be prepared to adopt healthy behaviors.

The proposed model has been designed to apply to the global health pandemic of COVID-19 and to home/community confinement with a number of important implications for policy makers to consider. The model is socially and economically favored for those whose confinement is not accompanied by loss of employment or threat to subsistence. Testing the model across different cultures and socio-economic contexts will help understand how different societies respond. Further, the framework is not limited to the Coronavirus SARS-2 and other health pandemics. It can be applied to different settings such as other places of confinement including hospitals, prisons and detention centers, rehabilitation centers, homes for the elderly and communities.

## Author Contributions

HR was the sole author of the manuscript, writing of whole manuscript, model development, editing and proof-reading.

## Conflict of Interest

The author declares that the research was conducted in the absence of any commercial or financial relationships that could be construed as a potential conflict of interest.
